# Individual Differences in the Speed of Facial Emotion Recognition Show Little Specificity but Are Strongly Related with General Mental Speed: Psychometric, Neural and Genetic Evidence

**DOI:** 10.3389/fnbeh.2017.00149

**Published:** 2017-08-10

**Authors:** Xinyang Liu, Andrea Hildebrandt, Guillermo Recio, Werner Sommer, Xinxia Cai, Oliver Wilhelm

**Affiliations:** ^1^State Key Laboratory of Transducer Technology, Institute of Electronics, Chinese Academy of Sciences Beijing, China; ^2^University of Chinese Academy of Sciences Beijing, China; ^3^Department of Psychology, Ernst-Moritz-Arndt-Universität Greifswald Greifswald, Germany; ^4^Differential Psychology and Psychological Assessment, Universität Hamburg Hamburg, Germany; ^5^Department of Psychology, Humboldt-Universität zu Berlin Berlin, Germany; ^6^Institute of Psychology and Education, Univeristy of Ulm Ulm, Germany

**Keywords:** face and object cognition, facial expression of emotion, processing speed, COMT val^158^met polymorphism, 5-HTTLPR/rs22531 polymorphism, event-related potentials

## Abstract

Facial identity and facial expression processing are crucial socio-emotional abilities but seem to show only limited psychometric uniqueness when the processing speed is considered in easy tasks. We applied a comprehensive measurement of processing speed and contrasted performance specificity in socio-emotional, social and non-social stimuli from an individual differences perspective. Performance in a multivariate task battery could be best modeled by a general speed factor and a first-order factor capturing some specific variance due to processing emotional facial expressions. We further tested equivalence of the relationships between speed factors and polymorphisms of dopamine and serotonin transporter genes. Results show that the speed factors are not only psychometrically equivalent but invariant in their relation with the Catechol-O-Methyl-Transferase (COMT) Val158Met polymorphism. However, the 5-HTTLPR/rs25531 serotonin polymorphism was related with the first-order factor of emotion perception speed, suggesting a specific genetic correlate of processing emotions. We further investigated the relationship between several components of event-related brain potentials with psychometric abilities, and tested emotion specific individual differences at the neurophysiological level. Results revealed swifter emotion perception abilities to go along with larger amplitudes of the P100 and the Early Posterior Negativity (EPN), when emotion processing was modeled on its own. However, after partialling out the shared variance of emotion perception speed with general processing speed-related abilities, brain-behavior relationships did not remain specific for emotion. Together, the present results suggest that speed abilities are strongly interrelated but show some specificity for emotion processing speed at the psychometric level. At both genetic and neurophysiological levels, emotion specificity depended on whether general cognition is taken into account or not. These findings keenly suggest that general speed abilities should be taken into account when the study of emotion recognition abilities is targeted in its specificity.

## Introduction

Human faces convey a large variety of socially relevant information. Face perception and emotion decoding (also termed perception, identification or recognition of emotions) are abilities of great importance in our everyday life. Despite a large amount of behavioral and neuroscientific research on face recognition and facial expression processing, comprehensive multivariate research, studying specificity with respect to individual differences across different levels of data, including behavior, neurophysiology and genetics, is still scarce. In the present work, we conceptually distinguish between measures of processing swiftness and processing accuracy, in line with a recent series of studies (e.g., Wilhelm et al., 2010; Hildebrandt et al., 2015). Focusing on speed measures, our overarching aim was to contrast the processing of socio-emotional (facial expressions of emotion), social (facial identity) and non-social (houses) stimuli from an individual differences perspective. We studied theoretically expected specificity of processing social and socio-emotional content at the level of multivariate ability measures, along with their genetic and neurophysiological correlates.

## Emotion Specificity Revealed by Psychometric Testing

Face processing, or more broadly spoken face cognition, refers to the perception of invariant facial features enabling the recognition of unfamiliar and familiar faces. Facial emotion perception, which goes beyond face identity processing, can be studied either as recognition ability of changes occurring in a given face regarding its expressive appearance, or as ability to distinguish the similarity of facial expressions across facial identities. The processing of the two types of facial information, identity and emotion expression, have been considered to rely on at least partly separable routes of the information processing system (Bruce and Young, 1986; Haxby et al., 2000).

The experimental and neuroscientific research on the commonalities and distinctions of facial identity and facial expression processing also informed individual differences studies. Wilhelm et al. (2010) called for a multivariate approach customary in intelligence research to address the specificity of face cognition-related abilities (see also Yovel et al., 2014; Lewis et al., 2016). In such an approach, an explicit distinction between easy and difficult tasks is crucial. Usually, individual performance differences during easy tasks—where accuracy is at ceiling—is reflected in response swiftness. In more demanding tasks, performance is usually measured in terms of accuracy. Thus, it is of great relevance to explicitly and systematically reflect on this distinction when developing psychometric tasks for measuring face cognition-related abilities (Wilhelm et al., 2010).

In a recent study, Hildebrandt et al. (2015) explored face identity and facial emotion processing based on measures of accuracy. The authors collected comprehensive accuracy data in a series of tasks intended to assess the perception and recognition of emotional facial expressions, perception and memory of face identity, along with general cognitive abilities measured by non-face tasks. The relationships between these variables were assessed via structural equation modeling (SEM). The results showed that the uniqueness of emotion perception accuracy is strongly limited, because 90% of the interindividual variance measured by facial emotion perception and recognition tasks could be explained as a multivariate linear function of face cognition and general cognitive abilities.

In previous work, processing speed, as measured in easy tasks, was also considered in estimating the distinction between facial identity and facial emotion processing. Hildebrandt et al. (2012) established a measurement model to investigate the relationships of speed abilities among facially expressed emotions, facial identity and non-face stimuli. The results showed no specificity for the speed of emotion recognition as compared with the speed of facial identity processing, and both abilities were moderately correlated with mental speed. These results are in line with another study reporting non-uniqueness of individual differences in the speed of cognitive processing measured in different content domains, comparing faces with non-face objects, that is, houses (Hildebrandt et al., 2013).

## Genetic Bases of Face Cognition Related Abilities

The specificity of social cognition can further be investigated at its genetic basis. One way of estimating heritability of traits is to study their association with single nucleotide polymorphisms. Here we first focus on the Catechol-O-Methyl-Transferase (COMT) val158met polymorphism, mainly studied in its association with general cognitive functioning. Second, we investigated the serotonin transporter-linked polymorphic region (5-HTTLPR), commonly related with emotion processing (von dem Hagen et al., 2011; Koizumi et al., 2013; Alfimova et al., 2015).

The COMT val158met polymorphism is known to play a role in cognitive abilities. The enzyme COMT degrades catecholamine neurotransmitters, including dopamine and epinephrine. The valine allele (Val) and the methionine allele (Met) are two identified variants of the COMT gene. The Met variant produces the enzyme with much lower activity than the Val variant, leading to higher dopamine concentrations in the synaptic cleft in carriers of the Met variant. Previous studies revealed the COMT val158met polymorphism to be correlated with general cognitive abilities (Kiy et al., 2013; Alfimova et al., 2015), with carriers of the Met allele usually outperforming Val+ carriers to a small degree.

The serotonin transporter (5-HTT) protein restricts serotonin transmission via reuptake from the synaptic cleft. The 5-HTTLPR is located in the promoter region of the 5-HT transporter (5-HTT) gene and shows a genetic polymorphism consisting in short (S) or long (L) allele variants, differing in their efficiency in producing 5-HTT and therefore in clearing the synaptic cleft from 5-HT. Carriers of the S allele have slower serotonin reuptake and higher serotonin concentrations in the cleft than carriers of the L allele (Lesch et al., 1996). Hu et al. (2006) reported single nucleotide variants (A and G) in the long allele, leading to a triallelic genotyping of 5-HTTLPR: S, L_A_ and L_G_. The 5-HTT protein transcription level of L_G_ is almost equivalent to S, both being lower than of the L_A_ genotype.

The 5-HTTLPR polymorphism has been related to emotion perception, both in healthy persons and patients with schizophrenia. L allele carriers have been reported to perform better than S carriers in emotion perception (Alfimova et al., 2015). 5-HTTLPR polymorphism has been also found to account for anxiety-related personality traits (Lesch et al., 1996) and to influence the sensitivity to positive and negative emotions (Koizumi et al., 2013). In a recent study, Hildebrandt et al. (2016) applied a latent variable modeling approach to test the discriminant relationship of the 5-HTTLPR polymorphism with facial identity and emotion perception vs. non-social cognitive abilities. By modeling fluid intelligence and immediate and delayed memory factors, along with face identity and facial emotion processing accuracy, the authors found the 5-HTTLPR/rs25531 polymorphism to be most strongly related with emotion processing abilities. This study supports a discriminant genetic basis of facial emotion perception, when performance accuracy is considered. However, it is still an open question whether facial emotion perception speed is also related with the 5-HTTLPR/rs25531 polymorphism and how it depends on stimulus content.

Furthermore, also using latent variable modeling to test discriminant relationships of the COMT val158met polymorphism with facets of cognitive abilities, Kiy et al. (2013) reported the COMT genotype to be related with general fluid abilities but not with face cognition ability after accounting for general cognition. Alfimova et al. (2015) also showed the COMT val158met polymorphism to be unrelated with emotion recognition in both, healthy persons and patients with schizophrenia.

## Neurophysiological Correlates of Face Cognition Abilities

Some specificity of face and facial emotion processing has been also demonstrated at the level of neurocognitive signals measured by event-related potentials (ERPs). ERPs consist of a sequence of components reflecting distinct cognitive processes, some of which are presumed to be sensitive to specific social or socio-emotional aspects of stimuli.

The P100 component is an early ERP deflection in response to visual stimuli. Though, usually viewed as a general component not related with the content specific relevance of the stimulus, it has been occasionally reported to be larger in response to faces than to other objects (e.g., Itier and Taylor, 2004; Thierry et al., 2007). Studies on emotion expression processing also revealed modulations of the P1, manifested by larger amplitudes as compared with neutral faces (Batty and Taylor, 2003). This effect was more obvious in some emotion categories in particular, for fearful and angry faces (Rellecke et al., 2012; Pourtois et al., 2013).

The N170 is a further early ERP, often considered as a marker of structural encoding of faces because it is larger in response to human faces as compared with non-face stimuli (Bentin et al., 1996; Itier and Taylor, 2004). Some studies revealed emotion-related modulations of the N170 component also (Blau et al., 2007; Lynn and Salisbury, 2008), manifested in a larger amplitude of N170 in response to emotional faces (especially fearful faces) when compared with neutral faces (Batty and Taylor, 2003; Rellecke et al., 2012).

Another ERP component commonly studied in conjunction with facial emotion processing is the early posterior negativity (EPN), defined as amplitude difference between ERPs to emotional and neutral faces within the time window of 200–350 ms over posterior electrodes. The EPN is larger for emotional than for neutral faces, and has been interpreted to reflect the enhanced sensory encoding of emotional relative to neutral expressions, driven by reflex-like attention to the stimulus (Schupp et al., 2004; Foti et al., 2009).

From an individual differences perspective, the above-mentioned ERP components have been recently studied with regard to their specific relationship with face identity and facial emotion perception accuracy. For example, latent variable analyses by Recio et al. (2017) revealed the latency of the N170 to be negatively related with the perception and recall of face identity, but not with emotion perception, whereas the EPN was related to both, face identity and facial emotion processing.

## Aims of The Present Study

As argued above, studies on the genetic and neurophysiological correlates of psychometric performance speed are missing. Only performance accuracy was investigated from an individual differences perspective along with its genetic and neurophysiological correlates. It remains unclear whether performance speed in emotion perception is related with specific gene polymorphisms and ERPs, as shown for performance accuracy.

Thus, in the present study we aimed to investigate the specificity of speed abilities for processing non-social, social and socio-emotional stimuli from an individual differences perspective. We focused on three different levels at which specificity may emerge: (1) psychometrics; (2) genetic associations; and (3) neurophysiological correlates. Our first goal was to assess whether individual differences in speed abilities are specific for different content domains: objects, faces with neutral expressions and facial emotion expressions. We applied multiple tasks of low difficulty in various stimulus categories and collected participants’ processing swiftness in all tasks. By using SEM, we investigated whether social and socio-emotional stimuli reveal systematic individual differences above a general factor of processing swiftness. In the light of previous research (Hildebrandt et al., 2012, 2016), we expected small or moderate specificity for processing emotion-related stimulus content.

Our second goal was to estimate the specificity of genetic relationships within the established structure of individual differences in speed-related abilities. We focused on polymorphisms associated with the serotonin and dopamine metabolism as two candidate genes affecting processing swiftness. We expected the COMT val158met polymorphism to be related with general processing efficiency, whereas the 5-HTTLPR/rs25531 polymorphism to be related with the processing of emotion-related content.

Finally, we aimed to investigate whether the P100, N170 and EPN components of the ERP wave are differentially related with different factors of processing speed. Because previous research revealed facial emotion processing to be substantially related with general cognition and face identity processing, we assessed genetic and neurophysiological relationships in two different scenarios: (1) When emotion related abilities are psychometrically modeled on their own; and (2) when emotion related abilities are nested under a general factor of processing swiftness of any kind of visually complex object. We expected the specific psychometrically captured variance of emotion processing swiftness to be associated with emotion related gene polymorphism and ERPs associated with emotion processing. Accordingly we postulated unspecific modeling to mask these relationships.

## Materials and Methods

### Participants

Volunteers were recruited for four testing sessions to collect psychometric data, and saliva samples for genetic analyses (session 1, 2 and 3), and EEG data for ERP analyses (session 4). A total of 273 healthy participants were enrolled in the psychometric part of the study. Their age ranged between 18 and 35 years. Four participants were excluded because they had missing values in more than five tasks due to technical problems and dropouts between testing sessions. The final sample consisted of 269 individuals (52% women), with a mean age of 26 years (*SD* = 5.92). These adults had heterogeneous educational backgrounds: 26.8% were not qualified by high-school education, 62.5% held high school degrees and 10.7% had academic degrees.

Saliva samples for genetic analyses could be collected from 230 persons (48% women). The average age of these participants was 25.9 years, *SD* = 4.5. Their educational background was as follows: 19.2% did not have a high-school degree, 49.2% had a high school degree, and 31.2% had acquired academic degrees. Among all participants, 87.2% were right-handed and 2.0% were ambidextrous. All participants reported normal or corrected-to-normal visual acuity.

For the EEG study, we randomly recruited 110 participants out of the psychometric sample, with sex and educational background distributed similarly to the original sample: 45.5% were females, the mean age was 26.5 years, *SD* = 4.8, 25.4% without high school degrees, 47.3% with high school degrees and 27.3% with academic degrees. Participants with error rates >30% during the emotion classification tasks or excessive EEG artifacts were removed from the analyses. The final sample in the EEG study included* n* = 102 participants (46 women), with a mean age of 26.64 years (*SD* = 4.82). These EEG data were also analyzed by Recio et al. (2017). However, Recio et al. (2017) investigated the relationship with these electrophysiological data and performance accuracy data that are not targeted in the present article. Also, the present research questions clearly differentiate from those asked in the previous work, in which performance speed and genetic variables were not targeted.

To summarize, the data available at different levels of measurement resulted in two subsamples. The maximal number of participants was *n* = 269 in the psychometric study. A subsample of *n* = 230 persons was also available in the genetic study, and a subsample of *n* = 102 in the EEG study. The EEG subsample was randomly drawn from the original psychometric sample, and was considerably fewer subjects because of resource limitations. Therefore, the number of participants for establishing the psychometric model was 269, for testing gene-ability associations was 230, and for testing brain-ability associations was 102. Because the EEG sample was smaller, we reduced the number of psychometric tasks when modeling brain–behavior relations (see below).

The present study conformed to the guidelines of the ethics committee of the Department of Psychology, Humboldt-Universität zu Berlin and the German Psychological Association. All participants signed informed consent before participating in the experiments in accordance with the Declaration of Helsinki. The protocol was approved by the ethics committee of the Department of Psychology, Humboldt-Universität zu Berlin (approval number 2012-46).

### Stimuli, Apparatus and Procedure

#### Psychometric Session

The psychometric study consisted of three sessions, each taking 3 h, separated by short intervals of 4–7 days. After a general introduction, participants completed a demographic questionnaire. Then, 18 tasks—described below—were administered across different content domains, including object, face and facial emotion processing, along with further measures of general cognitive functioning. After some practice trials with feedback on performance and clarification of any remaining questions, in all tasks participants were instructed to respond as quickly and accurately as possible without any feedback. Tasks were administered in a fixed sequence to all participants.

All tasks were programmed in Inquisit 3.2 (Millisecond Software, Seattle, MA, USA) software. Stimuli were presented on 17″ computer monitors with a resolution of 1680 × 1050. All facial emotion stimuli are described in Wilhelm et al. (2014).

#### EEG Session

In the EEG study, participants performed an expression identification task with dynamic stimuli, because such stimuli elicit larger ERP responses than static pictures (e.g., Recio et al., 2011). Emotion classification referred to six facial expressions: anger, disgust, fear, happiness, sadness and surprise, along with two neutral facial movements, blinking and chewing. The dynamic facial expressions of emotion were displayed with moderate and full intensities. Static face stimuli from the Radboud Faces Database (Langner et al., 2010) were morphed using FantaMorph (Abrosoft, 2010) to create dynamic expressions changing from neutral to emotional. Half of the expressions showed intermediate emotion intensities in order to increase task difficulty (Suzuki et al., 2006). Face stimuli were framed by an oval dark gray mask to hide any face-external features such as hair and neck and were shown as a color video format on a dark gray screen.

Each trial of the emotion classification task lasted for 1.3 s in total. After a 700-ms fixation cross, each stimulus started with a neutral expression and steadily increased to the maximal emotion intensity within 200 ms. The peak expression remained on display for another 400 ms. The onset of neutral face trials was also a neutral face, followed by a chewing or blinking movement shown for 200 ms. Then the stimulus returned to the initial state for 400 ms. In each trial, participants needed to choose among one of seven verbal emotion labels shown on the screen by mouse click; there were no time constraints.

There were 14 conditions (seven expressions by two intensity levels) in the emotion classification task, with 57 trials for each condition. The trials were presented in random order across conditions, but every participant received the same randomized sequence. Participants could take a short break after every 200 trials.

#### Genetic Analyses

The DNA analyses corresponded to those described in Kiy et al. (2013) and Hildebrandt et al. (2016). We extracted the DNA from buccal cells based on a method reported by Schonlau et al. (2010). Genomic DNA was automatically purified by using a commercial extraction kit (MagNA Pure LC DNA Isolation Kit; Roche Diagnostics, Mannheim, Germany).

To carry out genotyping for the COMT Val158Met polymorphism (rs4680), a real-time polymerase chain reaction (PCR) was performed by fluorescence melting curve detection analysis in the Light Cycler System (Roche Diagnostics). The advantage of melting curve analysis is that single-nucleotide polymorphisms (SNPs) can be detected without using the gel electrophoreses or sequencing followed after amplification. For the COMT/rs4680, the primer sequences of hybridization probes (TIB MOLBIOL Berlin, Germany), and the PCR protocol were as follows (Reuter et al., 2006): forward primer: 50-GGGCCTACTGTGGCTACTCA-30; reverse primer: 50-GGCCCTTTTTCCAGGTCTG-30; anchor hybridization probe: 50-LCRed640-TGTGCATG CCTGACCCGTTGTCA-phosphate-3; sensor hybridization probe: 50-ATTTCGCTGGCATGAAGGACAAG-fluorescein-30. The three genotypes are Val/Val, Val/Met and Met/Met. The COMT/rs4689 genotype distribution across participants was as follows: 49 persons were Val/Val (21.3%), 116 were Val/Met (50.4%) and 65 were Met/Met (28.3%) carriers.

The PCR method was also used for 5-HTTLPR/rs22531 genotyping. The MSP1 (New England Biolabs) was used to digest the PCR product and then incubated at 37°C for 1.5 h (Mastercycle, Eppendorf). The primers were as follows: 5-HTT-Msp-forward: tcc tcc gct ttg gcg cct ctt cc; 5-HTT-Msp-reverse: tgg ggg ttg cag ggg aga tcc tg. Followed by enzymatic digestion, gene samples were loaded onto a 1.6% agarose gel in a TBE solution, and run for 1 h 20 min at 170V. Subsequently they were visualized under UV light with the help of ethidiumbromide. Two different raters completed the genotyping of the samples by visual observation. They also repeated the operation on 20% of the samples, reaching a 100% concordance.

The 5-HTTLPR/rs22531 genotypes were labeled based on their transcriptional efficiency (Hu et al., 2006). There were three genotypes in total: the first was S’S’ (low activity), including S/S (14.4%), S/L_G_ (7.8%) and L_G_/L_G_ (0.4%). The second was L’S’ (intermediate activity), including S/L_A_ (38.7%) and L_G_/L_A_ (29.6%). The third was L’L’ (high activity), which only included L_A_/L_A_ (29.6%). The percentages of participants with different 5-HTTLPR/rs22531 genotypes were as follows: 52 persons were S’S’ (22.6%), 110 were L’S’ (47.8%), and 68% were L’L’ (29.6%) carriers. The calculated genotype frequency was in Hardy-Weinberg-Equilibrium: *χ*^2^ = 0.424, df = 1, *p* = 0.515.

### Descriptions of the Psychometric Tasks

#### Mental Speed Tasks

##### Finding A’s (MS1)

In each trial, one German word was shown on the screen. Participants were instructed to quickly and accurately decide whether there was a letter “A” contained in the presented word or not. They reacted by pressing the left key to “Yes” and right key to “No” responses.

##### Symbol substitution (MS2)

In each trial, one symbol out of “?”, “+”, “%”, or “$” was presented in the center of the screen. Participants indicated the symbol by pressing corresponding arrow keys, with the upward-pointing key associated to “?”, the right-pointing key to “+”, the down-pointing key to “%” and the left-pointing key to “$”.

##### Number comparison (MS3)

Two series of numeric strings appeared in a row. Participants had to decide whether the two strings were exactly the same or differed in one-number. Responses were given by left (different) or right (same) button presses.

#### Speed of Object Cognition Tasks

##### Simultaneous matching of morphed houses (SOC1)

House stimuli consisted of either two identical or two slightly different houses which were presented in each trial. There was a 50% probability for each kind of trial. Participants indicated whether the displayed houses were identical or not.

##### House verification (SOC2)

House stimuli were shown one by one on the screen. Participants provided responses according to the window features of the presented house and indicated whether there were only windows with a rectangular form or also of other shapes (e.g., round).

##### Delayed non-matching to sample houses (SOC3)

A target house was first presented for 1 s. After a retention interval of 4 s, a pair of houses was presented, consisting of the previously presented stimulus and a new one. Participants indicated the new house by button press.

#### Speed of Face Perception Tasks

##### Simultaneous matching of upper face-halves (SFP1 and SFP2)

Faces were segmented horizontally about midway of the nose into upper and lower halves. In each trial, a pair of faces combined from different face identities was shown. Participants indicated whether the two upper face halves were the same. In 50% percent of the trials each, the face halves were aligned or non-aligned. In the latter case the left or right edges of upper face halves were placed at the noses of the lower face halves. Aligned and non-aligned task conditions were used as separate indicators in the psychometric modeling.

##### Simultaneous matching of morphed faces (SFP3)

In each trial, two faces were presented after being morphed from the same two parent faces. They were morphed to different degrees, leading to trials with very similar faces (50%) vs. clearly dissimilar ones. Participants provided a two-choice response according to the similarity in each pair.

##### Simultaneous matching of faces from different viewpoints (SFP4)

Two faces per trial were presented in the diagonal of the screen. One was displayed in frontal view and the other in a three-quarter view. Participants decided whether the faces displayed the same or different persons.

#### Speed of Face Learning and Recognition Tasks

##### Delayed non-matching to sample faces (SFLR1)

A target face was first shown on the screen. After a 4-s delay, a pair of faces was presented simultaneously, including the target face and a new face. Participants indicated which of them was new.

##### Recognition speed of learned faces (SFLR2)

At the beginning of a trial block, four faces were shown for 1 min to allow for robust encoding, followed by a delay of about 4 min. During this period, participants worked on four figural reasoning items. Subsequently, a recognition phase followed, with four learned faces and four new faces presented one at a time in random order. Participants were to indicate whether a presented face was familiar or not.

#### Speed of Emotion Perception Tasks

##### Emotion perception from different viewpoints (SEP1)

Two different faces of the same gender were shown simultaneously. One was presented in frontal and the other in a three-quarter view. Each face expressed one of six “basic” emotions. Participants indicated whether the facial expressions were the same or not.

##### Identification speed of emotional expressions (SEP2)

In each trial, a verbal emotion label selected from the six basic emotions was presented in the middle of the screen. Around this emotion label, four non-identical faces of the same sex with different emotional expressions were shown. Participants were asked to indicate the targeted emotion out of the four faces by pressing arrow keys correspondingly.

##### Emotional odd-man-out (SEP3)

Three same-sex faces from different identities showing two different emotional expressions were displayed in each trial. The expression displayed by the face in the middle of the row served as reference. One of the flanking faces displayed the same emotion and the other one showed a different one. Participants indicated the divergent expression—the odd man out.

#### Speed of Emotion Learning and Recognition Tasks

##### 1-back recognition speed of emotional expressions (SELR1)

In each experimental block, a series of 24 facial expressions of emotion displayed by the same person were presented one by one. Participants indicated whether the current expression on the screen was the same as the one presented one trial before.

##### Recognition speed of morphed emotional expressions (SELR2)

Each block started with a learning session, during which four morphed facial expressions of the same person had to be memorized. After learning, participants answered two items from a scale measuring extraversion. Following this short delay, the recognition phase started. Emotion expressions were presented one by one. Only half of them were targets and participants indicated for each facial expression whether it had been presented in the learning phase.

##### Delayed non-matching to sample with emotional expressions (SELR3)

A facial expression was presented for 1 s (prime). After a delay of 4 s, including a 500 ms mask and a 3.5 s black screen, the same expression along with another facial expression were shown simultaneously. Participants indicated which of the two expressions did not match the prime.

The Supplementary Material Appendix (see Appendix A) provides an overview of the tasks along with their measurement intention and indicator abbreviation.

### Data Processing

#### Psychometric Data

As described above, the psychometric study contained multiple speed tasks with stimuli from various content domains, including houses, faces, and facial emotion expressions, as well as words, symbols and numbers. We used inverted latencies as indicators for psychometric modeling. In the preprocessing of the speed data, reaction times shorter than 200 ms were removed and the intraindividual RT data were winzorized. Average inverted latencies (1000/reaction time) were calculated for all correct responses. These scores represent the number of correctly solved trials per second.

#### Psychophysiological Data

EEG signal was collected from 42 electrodes using the left mastoid as reference and filtered from 0.032 Hz to 70 Hz. After being filtered again offline through a low-pass filter (30 Hz, 24 db/oct), the EEG signal was transformed to average reference. We also recorded electrooculogram from below and lateral to the eyes. Independent component analysis was used to remove eye blinks and horizontal eye movements. The preprocessed signal was then segmented into epochs, starting from 200 ms pre-stimulus to 1000 ms after stimulus. In order to obtain ERPs, we averaged these epochs for each individual facial expressions, and intensity. Thus, 14 indicators per ERP parameter were gained for each participant, to be used in psychometric modeling. Amplitudes and latencies of the P1 and N170 components were obtained by searching the maximum (peak) during time intervals 80–150 ms at PO8 site for the P1, and 155 to 210 ms at the P10 electrode for the N170. The EPN component was calculated as the average signal from a group of 12 electrodes in the posterior scalp area (P7, P8, P9, P10, PO7, PO8, PO9, PO10, O1, O2, Oz, Iz) in the period from 220 ms to 400 ms.

#### Coding the Gene Polymorphism Variables

Group membership depending on participants’ genotype was dummy coded (see e.g., Cohen et al., 2003) for both, the COMT Val158Met and the 5-HTTLPR serotonin polymorphisms. Two dummy variables for each gene polymorphism were entered as predictors into the SEM. Since there were three allele combinations in both COMT Val158Met and the 5-HTTLPR serotonin polymorphisms, we used two dummy variables (C1 and C2) for each polymorphism. In the case of 5-HTTLPR serotonin polymorphism we selected L’L’ homozygotes as the reference group to be compared with the other two, because the L’S’ heterozygotes and S’S’ homozygotes were not expected to differ. Thus, the coding variable C1 represented the difference between L’L’ and L’S’, and C2 represented the difference between L’L’ and S’S’ genotype groups. In case of the COMT Val158Met polymorphism, we selected Met/Met homozygotes as the reference group. Thus, C1 in case of COMT coded the difference between Met/Met and Val/Met, and C2 coded the difference between Met/Met and Val/Val. In Table 1 we summarize the dummy coding applied for psychometric modeling.

**Table 1:** Dummy coding of the Catechol-O-Methyl-Transferase (COMT) Val158Met and the serotonin transporter-linked polymorphic region (5-HTTLPR) serotonin polymorphisms.

Serotonin	COMT	C_1__LL_LS C_1__MM_VM	C_2__LL_SS C_2__MM_VV
L’L’	Met/Met	0	0
L’S’	Val/Met	1	0
S’S’	Val/Val	0	1

*Note. C1_LL_LS, first coding variable comparing L’L’ vs. L’S’ carriers; C1_MM_VM, first coding variable comparing Met/Met vs. Val/Met carriers; C2_LL_SS, second coding variable comparing L’L’ vs. S’S’ carriers; C2_MM_VV, second coding variable comparing Met/Met vs. Val/Val carriers; L’L’, 5-HTTLPR serotonin genotype with two long alleles; L’S’, 5-HTTLPR serotonin genotype with a long and a short allele; S’S’, 5-HTTLPR serotonin genotype with two short alleles; Met/Met, COMT Val158Met genotype with two methionine alleles; Val/Met, COMT Val158Met genotype with one valine and one methionine allele; Val/Val, COMT Val158Met genotype with two valine alleles*.

In the psychometric models, we used these dummy variables to explore the genotype effects on latent performance factors. Their regression weights reflect the differences in latent factor means of the reference group and the comparison group coded with 1 in a specific variable. Since we expected the best performance in zero-coded reference groups for both COMT (Met/Met) and serotonin (L’L’) polymorphisms, we anticipated negative regression weights in all cases. Latent variables were standardized in all psychometric models. Thus, the regression weights of the included dummy variables can be interpreted as follows: they reveal the expected difference in standard deviation units on the latent variable between the two genotype groups contrasted by a given coding variable.

#### Statistical Analyses and Expectations in the Psychometric Models

We performed the data analyses in multiple steps. First, we estimated a series of psychometric models to test the specificity of speed abilities in different content domains. By gradually increasing the number of content specific first-order speed factors, we investigated whether the model fit increases by adding these factors to the general factor of processing speed. We started by modeling emotion perception above the general factor, followed by emotion learning and recognition, face perception, face learning and recognition and object cognition.

Second, we tested gene-behavior and brain-behavior relationships of emotion perception speed. To this aim, we separately added the dummy variables coding the genotype groups to the psychometric model of emotion perception speed, indicated by three tasks described above. We expected the COMT Val158Met and the 5-HTTLPR serotonin polymorphisms to be non-specifically related with emotion processing speed when modeled on its own. Following the same rationale, five further models tested the relationships of the behavioral latent factor emotion perception speed with latent ERP factors for P100 amplitude, P100 latency, N170 amplitude, N170 latency and the EPN.

Third, we tested gene-behavior and brain-behavior relationships of emotion perception speed, accounting for its variance shared with general processing speed of complex objects, including houses and neutral faces. To this aim a more complex psychometric model was related to the dummy variables coding genetic polymorphisms and to the latent ERP factors. In this model facial emotion perception speed was a specific first order factor below the higher order general factor. Here, we expected the 5-HTTLPR serotonin polymorphisms to be specifically related with the emotion factor, whereas the COMT Val158Met polymorphism should only be associated with the general processing speed factor. In case of ERPs only the EPN may be related to the specific factor—based on previous research mentioned in the introduction above.

We used SEM and estimated model fit by the chi-square value (*χ*^2^), the comparative fit index (CFI), the root mean square error of approximation (RMSEA), and the standardized root mean-square residual (SRMR; see Bollen and Long, 1993). Computation was carried out with the package lavaan (Rosseel, 2012) in the R statistical software environment (R Core Team, 2016).

## Results

### Measurement Models Testing Content-Related Specificity of Processing Speed

**Figure 1:**
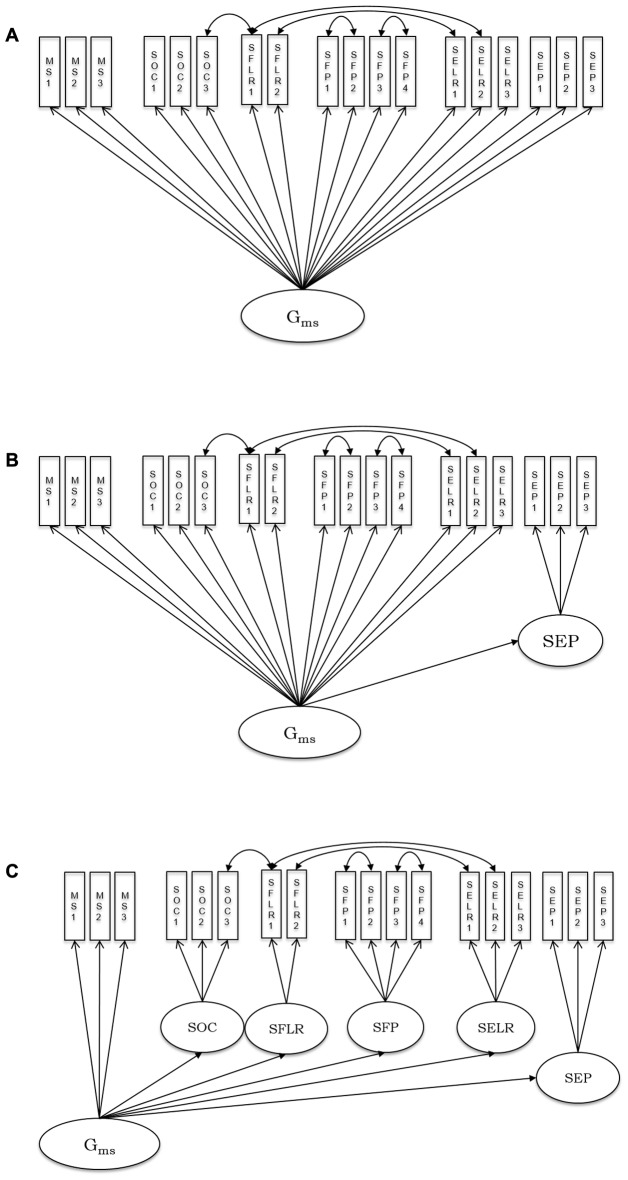
Schematic representation of the estimated psychometric models of processing speed including non-social, social and social-emotional stimuli. **(A)** Model 1; **(B)** Model 2; **(C)** Model 6; MS, mental speed; SOC, speed of object cognition; SFLR, speed of face learning and recognition; SFP, speed of face perception; SELR, speed of emotion learning and recognition; SEP, speed of emotion perception. See descriptions of all single indicators, along with the abbreviations used in the model graph in the “Materials and Methods” Section. Residual covariances were estimated between tasks sharing their procedure.

In order to support reanalysis of the present data in the SEM framework, the correlation matrices including all variables used for SEM in the present work and related sample information are available upon request from the senior author OW. As outlined above, we sequentially tested a series of models, starting with a general factor and adding specific content-related first-order factors one by one. Stepwise, models were inferentially compared. In Model 1, all observed speed variables from various psychometric tasks (see descriptions above) loaded onto a general cognitive speed factor (G_ms_). This model is depicted in Figure 1A. From Model 2–6, we sequentially added first-order factors representing different stimulus content categories: emotion perception speed (Figure 1B), emotion learning and recognition, face perception, face learning and recognition and object speed (Figure 1C).

In the first model, we tested whether a general speed factor exhaustively explained individual differences in processing all kinds of stimuli applied in the present tasks. The model assumes no substantial individual differences that are specific to content domains. The fit of this model indicated that there is room for improvement: $\chi_{\left(130\right)}^{2}$ = 296.29, *p* < 0.01, CFI = 0.94, RMSEA = 0.08, SRMR = 0.05. All factor loadings were significant and their standardized values ranged between 0.51 and 0.81.

Because we expected specificity for emotion processing, in Model 2 we added a first-order factor accounting for specific variance in the speed of emotion perception. This model showed a better fit to the data than Model 1: $\chi_{\left(129\right)}^{2}$ = 260.52, *p* < 0.01, CFI = 0.95, RMSEA = 0.07, SRMR = 0.04, and the improvement of fit as compared to the one-factor model was statistically significant: Δχ^2^ (Δ*df* = 1) = 35.77, *p* < 0.01. All factor loadings were statistically significant. Standardized loadings on the general factor ranged between 0.51 and 0.89. The three standardized loadings on the emotion-specific factor were: 0.73, 0.87, and 0.83. Thus, Model 2 shows statistically substantial specificity for the emotion-related factor above the general factor. Its loading on the general factor is however high, with a value of 0.89, and consequently. There is statistically significant residual variance of 21%-representing some, but limited emotion specificity of processing speed.

In Model 3, we further elaborated on Model 2 by adding another latent variable accounting for specificity in learning and recognition of emotional expressions. However, adding this factor led to model non-convergence due to a loading of the additional factor on the general one, which was above unity. Thus, we fixed the loading of the emotion learning and recognition factor on G_ms_ to 1, and continued the stepwise model comparison by adding a further factor in Model 4. The additional factor in Model 4 aimed to account for specificity in speed of face perception. The model with a specific emotion perception and a specific face perception factor converged, $\chi_{\left(128\right)}^{2}$ = 258.45, *p* < 0.01, CFI = 0.95, RMSEA = 0.07, SRMR = 0.04; but there was no significant improvement in fit for Model 4 above Model 2: Δχ^2^ (Δ*df* = 1) = 2.07, *p* = 0.15. In Model 5 we added face learning and recognition speed as a specific factor, but the model did not converge. The reason was again a perfect relationship between the face learning and recognition factor with the general factor. Finally, Model 6 included a factor specific for object cognition but revealed no better fit as compared with Model 2: Δχ^2^ (Δ*df* = 1) = 3.83, *p* = 0.05.

Based on the specified model series, we can conclude that Model 2, including a general cognitive speed factor and a first-order emotion perception factor, is the most parsimonious and best fitting model describing individual differences in the speed of processing non-social, social and socio-emotional stimuli. This model revealed some emotion perception-related specificity above general performance speed, with a residual variance of 21% indicating the extent of specificity.

Although not a main aim of the present study, sex differences are commonly of interest in emotion research. Therefore, we additionally studied the difference between female and male participants regarding both, general and emotion-specific performance speed. To this aim we regressed the two speed factors estimated in the final psychometric model (Model 2) into a dummy coded variable representing sex differences. Women were chosen as the reference group. The fit of this model was acceptable: $\chi_{\left(145\right)}^{2}$ = 288.46, *p* < 0.01, CFI = 0.95, RMSEA = 0.07, SRMR = 0.05. Although women tend to show a slight advantage in both performance domains, there were no statistically substantial sex differences either in general processing speed (*β* = −0.20, *p* = 0.15), nor at the level of the emotion-specific speed factor (*β* = −0.11, *p* = 0.56). Next, we tested whether genetic and neurophysiological correlates of the psychometrically specific emotion processing factor that can be generalized across sex, are also distinct.

### Gene Polymorphisms and Emotion Perception Speed

To study the differences between genotype groups in emotion perception speed, we extracted this factor from the psychometric Model 2 described above. Note that our first aim was to test genetic relationships in a scenario where the phenotype is not yet modeled as a specific factor within the nomological net of related abilities. Emotion perception speed, indicated by three different tasks was regressed into the dummy variables coding genotype groups of the COMT Val158Met and the 5-HTTLPR serotonin polymorphisms. These two genetic polymorphisms were considered in separate models (Table 2). Model fits were acceptable in both cases. The COMT Val158Met polymorphism was related with emotion perception speed, showing the Met/Met genotype group to perform significantly better than Val/Met genotype group (*β* = −0.35, *p* = 0.04) and as suggested by the effect size, somewhat better than Val/Val genotype group (*β* = −0.25, *p* = 0.22). However, the serotonin genotype groups did not differ in their emotion processing ability, suggesting that—when the phenotype is not modeled as a specific factor within the nomological net—genetic relationships are not emotion-specific.

**Table 2:** Relationships between the emotion perception speed factor and genotypes along with the fit of the models in which these relations have been estimated.

Gene	Coding variables	*β*	*p*	χ^2^(*df*)	CFI	RMSEA	SRMR
COMT	C1_MM_VM	−0.35*	**0.04**	0.46 (4)	1.00	0.00	0.01
	C2_MM_VV	−0.25	0.22			
Serotonin	C1_LL_LS	−0.07	0.67	3.83 (4)	1.00	0.00	0.02
	C2_LL_SS	−0.06	0.75			

Note. C1_MM_VM, first coding variable comparing Met/Met vs. Val/Met carriers; C2_MM_VV, second coding variable comparing Met/Met vs. Val/Val carriers; C1_LL_LS, first coding variable comparing L’L’ vs. L’S’ carriers; C2_LL_SS, second coding variable comparing L’L’ vs. S’S’ carriers. *p < 0.05; bold values represent significant results.

### ERP Correlates of Emotion Perception Speed

**Figure 2:**
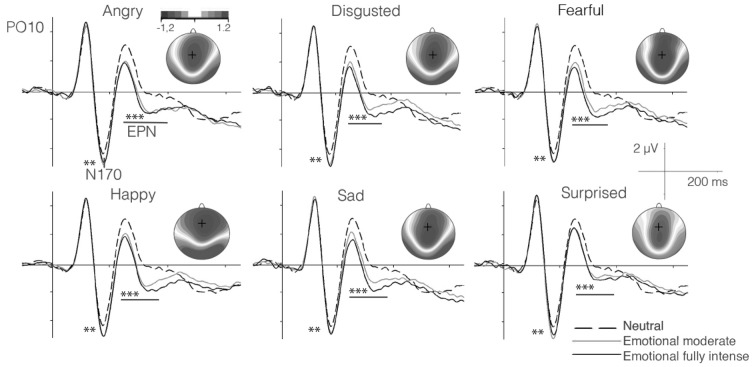
Grand average event-related potentials (ERPs) for neutral (chewing) as compared to high- and moderate-intensity dynamic emotional movements for each basic emotion in the subsample of the EEG study (*n* = 102). Significant effects for the difference in amplitude between emotional and neutral expressions for the N170 and early posterior negativity (EPN) components are marked by asterisks (Note: **p < 0.01; ***p < 0.001. Topographies show the amplitude effects of high intensity emotion over neutral conditions during the time segment 220–400 ms. The present EEG data were also analyzed by Recio et al. (2017), however with clearly distinct aim. Figure 2 is similar, but in its details distinct from the Figure provided in the previous work.

As described above, for each participant, we parameterized the P100, N170 and the EPN components to study their relationships with the speed of emotion perception ability. The N170 and EPN components are displayed as grand averages in Figure 2, along with the topographies visualizing the EPN in different emotion conditions. For psychometric modeling, each component was parameterized across trials, separately for each participant and each emotion category and neutral conditions (see above).

**Figure 3:**
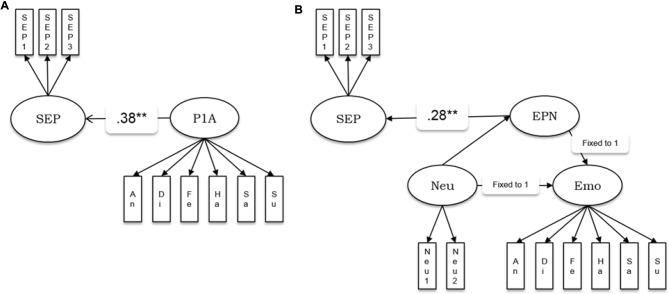
Schematic representations of the structural models estimating the relationship between ERP components and the speed of emotion perception. **(A)** P1A—amplitude of the P100 component. This model structure was also applied to the P100 latency, the N170 amplitude and the N170 latency. **(B)** EPN amplitude as latent difference score (LDS) related to the speed of emotion perception. SEP, Speed of Emotion Perception; P1A, P100 amplitude, An, Di, Fe, Ha, Sa, Su, Neu, faces expressing anger, disgust, fear, happiness, sadness, surprise and no emotion, respectively; EPN, Early Posterior Negativity; Neu, latent variable estimated based on neutral indicators; Emo, latent variable estimated based on emotion specific indicators. **p < 0.01.

We estimated the relationships of the amplitudes and latencies of the P100 and N170 components, as well as the EPN amplitude with the behavioral factor for the speed of emotion perception measured independently in the psychometric sessions. We estimated five separate models in which the speed of emotion perception was regressed into a latent factor representing amplitudes or latencies of the ERP components (see Figure 3 as example of the model for the P100 amplitude and the EPN). The model in Figure 3A was applied to the amplitudes and latencies of both P100 and N170. A latent variable defined by six emotion category-specific indicators (e.g., P100 amplitude for faces showing anger, disgust, etc.) represented the ERP component. The latent variable speed of emotion perception was regressed into the latent ERP variable.

Because the EPN component is defined as the amplitude difference between emotional and a neutral stimuli conditions, the measurement model of the EPN used latent difference score (LDS) modeling (e.g., McArdle, 2009), with a similar approach as in Recio et al. (2017). As visualized in Figure 3B, the EPN is estimated as the latent difference in ERP amplitude between all conditions showing facial expressions of emotion and the neutral baseline conditions (chewing and blinking movements). In this model, the variance across persons in the emotion condition is decomposed into the variance of the amplitude in the EPN interval, measured during the neutral face identification vs. the difference between emotion and neutral conditions. This decomposition of variance can be achieved by fixing two regression paths directed to the variable to be decomposed to unity (see e.g., McArdle, 2009; Kaltwasser et al., 2014; Recio et al., 2017). All regression weights parameterizing the relationship between the ERPs and the speed of emotion perception, along with model fit parameters, are provided in Table 3.

**Table 3:** Relationships between the speed of emotion perception and event-related potential (ERP) components.

ERP	χ^2^(*df*)	CFI	RMSEA	SRMR	*β*	*p*
P1A	42.44 (26)	0.99	0.08	0.01	0.38**	**0.001**
P1L	41.15 (26)	0.99	0.08	0.06	−0.01	0.92
N170A	36.24 (26)	0.99	0.07	0.05	0.16	0.16
N170L	15.42 (26)	1.00	0.00	0.02	−0.22	0.06
EPN	39.44 (42)	1.00	0.00	0.04	0.28*	**0.02**

Note. P1A, P100 amplitude; P1L, P100 latency; N1A, N170 amplitude; N1L, N170 latency; EPN, EPN amplitude; β, regression weight of the emotion perception factor into the ERP factors. *p < 0.05, **p < 0.01. Bold values represent significant results.

Results in Table 3 show very good fits for all models. The swiftness of emotion perception was significantly related only with P100 amplitude (*β* = 0.38, *p* < 0.01) and EPN amplitudes (*β* = 0.28, *p* = 0.02). Additionally, the size of the relation between N170 latency and the swiftness of emotion perception suggests a small effect, which however did not reach statistical significance.

### Genetic Correlates of Emotion Specific Perception Speed after General Speed Was Accounted for

In the next step, we estimated genetic correlates of specific individual differences in processing emotional faces because relating specific variance components of the phenotype measures to the genotype variables may reveal non-generic genetic bases of specific ability estimates. To estimate specific genotype effects, we partialled out the shared variance of emotion perception speed with general processing speed related abilities, as established in the psychometric Model 2 depicted above. Estimated from Model 2, we regressed both, the general speed factor G_ms_ and the emotion perception speed factor onto the dummy variables coding genotype groups. The fit of Model 2, additionally including the COMT gene was good: $\chi_{\left(161\right)}^{2}$ = 288.65, *p* < 0.01, CFI = 0.95, RMSEA = 0.06, SRMR = 0.05. The same was true for the model including the serotonin gene: $\chi_{\left(161\right)}^{2}$ = 283.38, *p* < 0.01, CFI = 0.95, RMSEA = 0.06, SRMR = 0.05. The gene-behavior relationships provided by these two models are summarized in Table 4.

**Table 4:** Relationships between general processing speed and the specific emotion perception speed factors and genotypes.

	C1_MM_VM		C2_MM_VV		C1_LL_LS		C2_LL_SS	
	*β*	*p*	*β*	*p*	*β*	*p*	*β*	*p*
G_ms_	−0.540**	0.001	**−0.380**	0.058	−0.118	0.467	0.148	0.444
SEP	0.272	0.253	0.188	0.507	0.095	0.680	**−0.425**	0.127

Note. G_ms_, general mental speed factor; SEP, speed of emotion perception factor; C1_MM_VM, first coding variable comparing Met/Met vs. Val/Met carriers; C2_MM_VV, second coding variable comparing Met/Met vs. Val/Val carriers; C1_LL_LS, first coding variable comparing L’L’ vs. L’S’ carriers; C2_LL_SS, second coding variable comparing L’L’ vs. S’S’ carriers. To graphically visualize the main findings depicted in this table we estimated factor scores for general processing speed and the specific emotion perception speed factors. Boxplots for the contrasts depicted in this table are displayed in the Supplementary Material Appendix (see Appendix B). Please note that factors scores do not completely retain the model structure, and thus, latent level correlations estimated in the model do not always perfectly match relationships with factor scores which was the case for the contrast C1_LL_SS. **p < 0.01. Bold values represent significant results and strong correlations.

As indicated in Table 4, the general speed factor was related with the COMT Val158Met polymorphism, showing that the Met/Met genotype group performed half a standard deviation better as compared with the heterozygotes, and above a third of a standard deviation as compared with the Val homozygotes. There was no relationship between the serotonin polymorphism and the general speed performance. However, the serotonin analyses revealed a specific relationship with emotion perception speed, showing that the L’L’ group performed almost half of a standard deviation better as compared with the S’S’ group. Due to the limited variance of the emotion-specific factor and the moderate power for the genetic analyses, this effect did not reach statistical significance, even though the effect size is considerable for a single polymorphism effect.

### ERP Correlates of Emotion Perception Speed after General Speed Was Accounted for

Finally, to study the specificity of emotion perception speed and its relations with ERP components, we related the variables in the psychometric Model 2 with the P100 amplitude and latency, the N170 amplitude and latency and the EPN. The measurement models for the ERPs were the same as above, when relating them to emotion perception modeled as single latent variable outside its nomological net. Because the subsample of the EEG study was limited to 102 participants, we reduced the number of indicators in the psychometric model of behavior. The general factor was only indicated by the three mental speed tasks and the three object cognition tasks in this reduced model. The fit—as depicted in Table 5, along with brain-behavior relationship—was acceptable in case of all structural models. All ERP components, except the P100 latency, were substantially related with the general, but not the emotion specific factors, suggesting a lack of emotion processing-related specificity at the level of neurophysiological correlates.

**Table 5:** Relationships between the general speed and the speed of emotion perception as specific factor and ERP components.

ERP	χ^2^ (*df*)	CFI	RMSEA	SRMR	*β*_Gms_	*p*	*β*_SEP_	*p*
P1A	116.87 (87)	0.98	0.06	0.05	0.27*	**0.02**	−0.16	0.06
P1L	130.52 (87)	0.97	0.08	0.07	0.06	0.62	−0.08	0.32
N170A	131.81 (87)	0.98	0.08	0.08	0.26*	**0.02**	0.10	0.23
N170L	99.22 (87)	0.99	0.04	0.06	−0.26*	**0.03**	0.01	0.89
EPN	134.68 (115)	0.99	0.04	0.04	0.31*	**0.01**	−0.01	0.86

Note. P1A, P100 amplitude; P1L, P100 latency; N1A, N170 amplitude; N1L, N170 latency; EPN, EPN amplitude; β_Gms_, standardized regression weight of the general cognitive speed factor onto the ERP factors; β_SEP_, standardized regression weight of the emotion-specific speed factor onto the ERP factors. To graphically visualize the main findings depicted in this table we estimated factor scores for the general processing speed factor. Scatterplots for the relationship with P1A, N170A, N170L and EPN depicted in this table are presented in the Supplementary Material Appendix (see Appendix C). Please note that factors scores do not completely retain the model structure, and thus, latent level correlations estimated in the model do not always perfectly match relationships with factor scores. They are provided for visualization purpose only. Inferential tests are model based. *p < 0.05. Bold values represent significant results.

## Discussion

The challenge to account for individual differences in abilities at the genetic and neurophysiological levels is not only to adequately measure the biological variables, but also to develop a sophisticated understanding of behavioral ability measures, thus of the phenotypes. Although plenty of research made considerable efforts on the first, works including sound psychometric modeling of multiple indicators of cognitive performance and behavior, as done here, are rather rare. In correlative or quasi-experimental studies—as unavoidable in investigating biological correlates of individual differences in humans—it is decisive to elaborate explanatory models of the captured performance. Whenever abilities are investigated, this is particularly important because human abilities show ubiquitous positive manifold. In other words, unequivocal interpretations of relations between biological and behavioral measures require explicit consideration of collinearities and the hierarchical structure of abilities. Indeed, the collinearities between various ability measures might be so high, that there is little to no specificity of a specific task class. If such collinearity is not considered by explanatory psychometric models, conclusions derived for biological covariates may be flawed.

In the present study, we first estimated individual differences in cognitive speed-related abilities across a series of stimulus domains, including non-social, social and socio-emotional contents to explore the psychometric structure of these abilities. Our analyses revealed some specificity for processing facial expression of emotion. Second, we estimated a series of gene-behavior relationships to study emotion specificity of processing speed. When emotion perception was modeled as a specific factor in its nomological net, emotion specificity was associated with serotonin availability, whereas dopamine availability was related to general speed of processing. Third, we studied brain-behavior relationships between behavioral factors and ERP components measured during an emotion classification task. We expected the P100 and N170 components to be related with general processing speed of faces and objects, and the EPN to be related with emotion processing speed. Brain-behavior relations were not specific for emotion. To study how the specific modeling of phenotypes influence whether gene-behavior and brain-behavior relations can be uncovered, we estimated all these relations under two conditions: we first related emotion perception speed with genes and ERPs when this ability was psychometrically modeled on its own, and second, when emotion perception speed was modeled in its nomological net as a specific factor showing some specificity above general processing speed. In the following, we discuss implications of our findings regarding emotion specificity of processing speed, as revealed by psychometric, gene-behavior and by brain-behavior relationships analyses.

### Emotion Specificity at the Psychometric Level

Although individual differences in face processing accuracy have been shown not to be equivalent with object cognition (Wilhelm et al., 2010; Hildebrandt et al., 2011, 2015), speed abilities of processing non-social, social and socio-emotional stimuli did not show significant factorial distinctions in previous work (Hildebrandt et al., 2012, 2016). In the psychometric analyses presented in this study, we partly replicated previous reports. All latent factors representing speed abilities in different content domains were highly related with the general speed factor. Only the emotion perception factor showed some specific variance.

Our psychometric results complement previous studies about the specificity of face and facial expression processing (Wilhelm et al., 2010; Hildebrandt et al., 2012, 2015, 2016). Wilhelm et al. (2010) emphasized the importance of considering speed and accuracy measures as two separate facets also of face cognition-related abilities when studying their specificity within the structure of cognitive abilities. Face cognition was shown to be distinguishable from general cognition at the level of accuracy measures in difficult tasks (Wilhelm et al., 2010; Hildebrandt et al., 2011) but not in the speed of processing easy tasks (Hildebrandt et al., 2012, 2016). The accuracy of facial emotion perception and memory accuracy was specific above general cognition, but when additionally considering face cognition accuracy emotion specificity in performance accuracy was keenly restricted (Hildebrandt et al., 2015). The present study was the first to investigate emotion-related specificity of performance speed using complex objects (houses) and neutral faces simultaneously. The results indicate moderate emotion specificity in speed performance as compared with general cognitive abilities, mental speed, object cognition and face identity cognition. We assume this specificity to be due to demands of emotion-related activation. For face identity processing, the psychometric picture is more complex: accuracy measures are strongly specific, whereas speed measures are not at all.

The content-independency of speed abilities might be related with the connectivity of the complete brain structure. In a neuro-anatomical study on general intelligence, including mental speed, Penke et al. (2012) applied quantitative tractography to measure the relationships between general intelligence, mental speed and three white matter integrity biomarkers, namely fractional anisotropy (FA), longitudinal relaxation time (T1) and magnetization transfer ratio (MTR). The mental speed factor was modeled as an intermediate factor between white matter integrity and general intelligence. The results provided evidence that lower brain white matter tract integrity had a negative influence on general intelligence, which was however mediated through processing speed. These results by Penke et al. (2012) let us assume that the connectivity of the whole brain may generate individual differences in processing speed, whereas content specific individual differences may be rather due to brain structure characteristics in dedicated brain areas.

### Emotion Specificity at the Level of Gene-Behavior Relationships

Based on Tables 2, 4, we may conclude that the dopamine-related COMT is associated—as expected—with the general speed factor, whereas the serotonin related polymorphism was associated with the specific variance in emotion perception after general speed was accounted for. Please note that the serotonin effect remained undetected when general speed was not taken into account by modeling individual differences in the phenotype.

As a facet of face cognition, facial emotion perception accuracy has been reported to have specific genetic covariates. Emotion perception accuracy is more strongly related with the 5-HTTLPR serotonin transporter polymorphism as compared with face identity perception and memory, as well as with processing non-social stimuli (Adayev et al., 2005; Hildebrandt et al., 2016). On the other hand, the COMT val158met polymorphism was reported to be associated with a series of cognitive performance variables, such as fluid cognitive abilities and working memory (e.g., Aleman et al., 2008; Kiy et al., 2013). The present study revealed: when emotion perception speed is considered, only the specific variance due to emotion, after general speed was controlled for, uncovered relations with the gene regulating serotonin availability. This novel finding suggests that speed abilities are strongly interrelated, but some genetic covariates seem to be rather specific.

When we study emotion perception speed without considering the nomological net around it, this ability was related with the COMT gene polymorphism regulating dopamine, but there was no correlation with the gene responsible for regulating serotonin. This finding is interesting in at least two regards. First, it suggests that emotion perception speed is a cognitive ability obviously facilitated by dopamine availability in the prefrontal cortex, because the COMT gene has been shown to be related with dopamine availability. Second, the finding suggests that interindividual variance caused by differences in general processing speed suppresses the effect of serotonin availability on emotion perception speed. Only if the phenotype is specifically modeled, the serotonin effects emerged, whereas the COMT gene polymorphism was still related with the general cognitive speed factor. These findings convincingly support a more general genetic influence on emotion perception triggered by the fact that the perception of emotional expressions is obviously a cognitive ability with a specific genetic basis.

These findings are novel in two respects: first, there is no study on the genetic bases specifically of facial emotion processing speed, and second, there is no study showing differential relationships of genes regulating dopamine vs. serotonin. Future studies might build up on these findings, possibly including epigenetics and interactions with environmental factors.

### Emotion Specificity at the Level of Brain-Behavior Relationships

When emotion perception is modeled outside its nomological net, the following conclusions can be drawn about the brain-behavior relationships: quicker emotion perception performance is reflected by higher amplitudes of the early P100 component and a stronger modulation of the ERP in the time range of the EPN in case of emotion stimuli as compared with neutral ones. However, when we partialled out the shared variance with the general speed factor, the P100, the N170 and the EPN components revealed no specificity of emotion perception speed. This was expected for the two early components in our explicit emotion categorization task where emotion effects typically start after the N170 component (e.g., Schacht and Sommer, 2009; but see Wang and Li, 2017, for divergent results from an implicit task), but not for the EPN. Using a similar modeling approach, Recio et al. (2017) showed a valence-specific relationship between emotion perception accuracy and the EPN amplitude. Interestingly, they also found that the EPN elicited by non-emotional face movement was related with emotion perception, which argues against a strong emotion specificity of the EPN component.

Even if brain-behavior relationships did not indicate emotion specificity, we found a number of substantial associations at the level of general speed ability. Our study showed the overall swiftness of processing complex objects from several domains, including houses, faces and facial expressions to be associated with larger P100 and N170 amplitudes, shorter N170 latency and larger EPN.

To our knowledge, this is the first study reporting ERP correlates of emotion perception speed. The current findings complement recent findings indicating associations between the N170 latency and the accuracy of face perception and memory, and between the EPN amplitude and emotion perception accuracy (Recio et al., 2017).

## Conclusion

In summary, the present study not only further supported the limited uniqueness of general speed abilities across non-social, social and socio-emotional domains, but also it is the first to investigate the specificity of emotion processing speed by estimating its genetic and neurophysiological correlates by considering two different phenotype definitions. The COMT gene polymorphism was consistently related with the general speed factor, while the serotonin was related with the speed of emotion perception only when its shared variance with the general speed factor was partialled out. The brain-behavior analyses showed little emotion-specificity for the ERP correlates of emotion processing speed. But relationships were revealed between general swiftness and the P100, N170 and EPN components. Besides, strict definitions based on explanatory psychometric models of multiple behavioral indicators are necessary, because different variance components captured by a given measure of a cognitive phenotype may lead to very different analysis outcomes due to the positive manifold or even high collinearity that characterize cognitive phenotypes.

## Author Contributions

AH, GR, WS and OW designed the study. They also supervised and conducted data aquisition. GR parameterized ERP data. XL and AH analyzed the data. XL, OW, AH and WS conceptually set up psychometric models. XC supervised and advised XL in data analyses. XL drafted the manuscript, which was edited by all co-authors in several revision stages.

## Conflict of Interest Statement

The authors declare that the research was conducted in the absence of any commercial or financial relationships that could be construed as a potential conflict of interest.
