# Supplementary material for: Individual Differences in the Speed of Facial Emotion Recognition Show Little Specificity but Are Strongly Related with General Mental Speed: Psychometric, Neural and Genetic Evidence
Source: Front Behav Neurosci. 2017 Aug 10;11:149. doi: 10.3389/fnbeh.2017.00149 (PMC5554373; doi:10.3389/fnbeh.2017.00149)

**Appendix A: Overview of the tasks and performance indicators**

| **Ability domain** | **Task-Number** | **Name of the task (Abbreviation of indicator)** |
| --- | --- | --- |
| Mental Speed | 1 | Finding A’s (MS1) |
|  | 2 | Symbol substitution (MS2) |
|  | 3 | Number comparison (MS3) |
| Speed of object cognition | 4 | Simultaneous matching of morphed houses (SOC1) |
|  | 5 | House verification (SOC2) |
|  | 6 | Delayed non-matching to sample houses (SOC3) |
| Speed of face perception | 7&8 | Simultaneous matching of upper face-halves (SFP1 and SFP2) |
|  | 9 | Simultaneous matching of morphed faces (SFP3) |
|  | 10 | Simultaneous matching of faces from different viewpoints (SFP4) |
| Speed of face learning and | 11 | Delayed non-matching to sample faces (SFLR1) |
| recognition | 12 | Recognition speed of learned faces (SFLR2) |
| Speed of emotion perception | 13 | Emotion perception from different viewpoints (SEP1) |
|  | 14 | Identification speed of emotional expressions (SEP2) |
|  | 15 | Emotional odd-man-out (SEP3) |
| Speed of emotion learning | 16 | 1-back recognition speed of emotional expressions (SELR1) |
| and recognition | 17 | Recognition speed of morphed emotional expressions (SELR2) |
|  | 18 | Delayed non-matching to sample with emotional expressions (SELR3) |

**Appendix B: Boxplots visualizing the main findings in Table 4**

*Note*. Boxplots are based on factor scores estimated for the latent variables G_ms_ and SEP. Please note that factor scores do not completely retain the model structure. Thus, latent level correlations with a grouping variable (gene polymorphism) estimated in the model do not always perfectly match the correlations between estimated factor scores. However, these plots are helpful to visualize the main results displayed in Table 4. We interpret the correlations from the latent variable model only and use the factor scores for visualization purpose. Left panel: COMT Met/Met and Val/Met contrast on G_ms_ (General mental speed); Right panel: Serotonin L’L’ and S’S’ contrast on SEP (Speed of emotion perception).


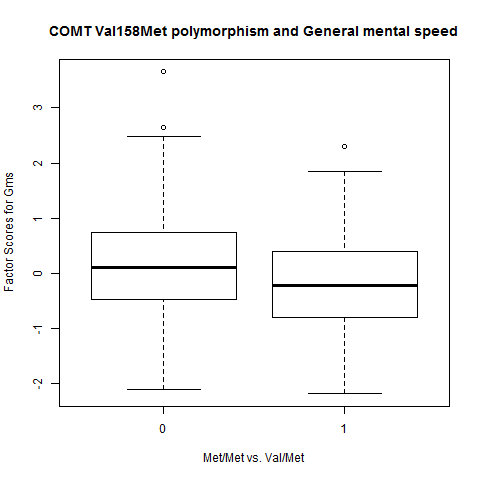

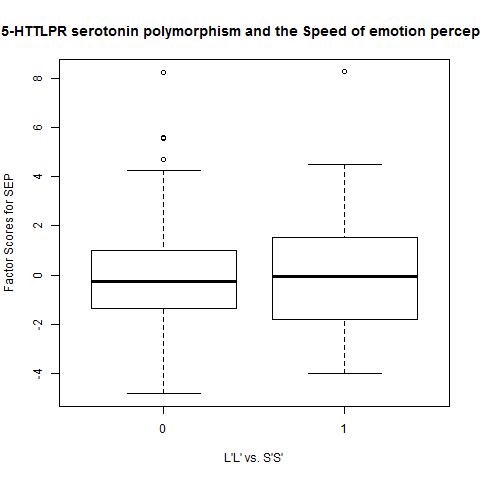


**Appendix C: Scatterplots visualizing the main findings in Table 5**

*Note*. Correlation plots are based on factor scores estimated for the latent variables G_ms_ and SEP in the models depicted in Table 5. Please note that factor scores do not completely retain the model structure. Thus, latent level correlations estimated in the model do not always perfectly match the correlations between estimated factor scores. However, these plots are helpful to visualize the main results displayed in Table 5. We interpret the correlations from the latent variable model only, but they are not substantially different from those of the factor scores plotted below. Top left panel: Relationship of general processing swiftness and the amplitude of the P100; Top right panel: Relationship of general processing swiftness and the latency of the N170; Bottom left panel: Relationship of general processing swiftness and the amplitude of the N170; Bottom right panel: Relationship of general processing swiftness and the EPN.


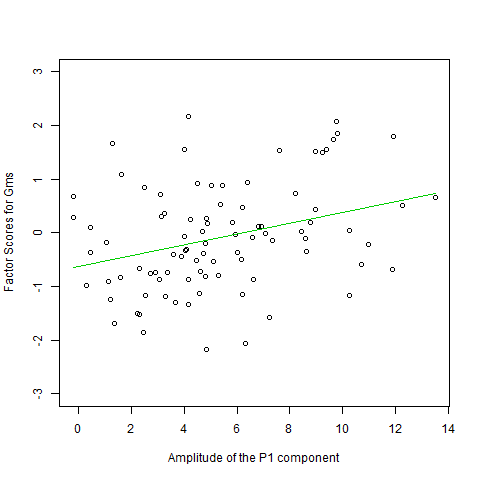

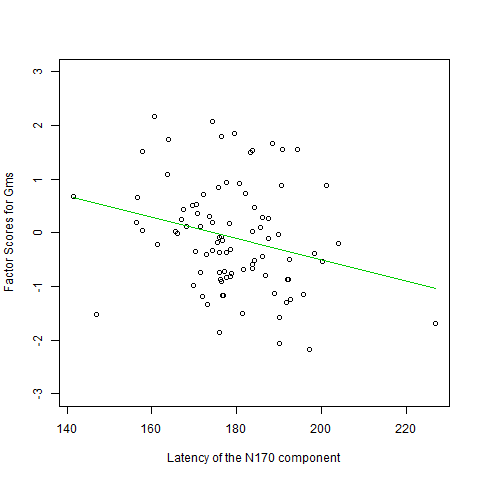


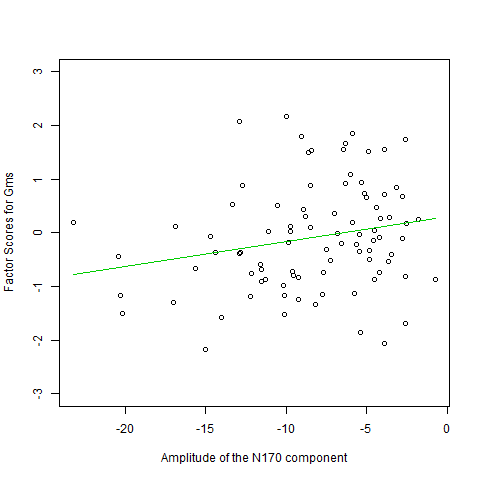

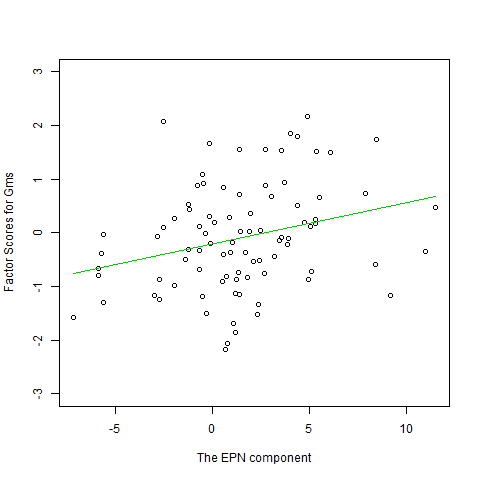

Supplement: Supplementary file 1 [file Data_Sheet_1.docx]
